# PRNP Haplotype Associated with Classical BSE Incidence in European Holstein Cattle

**DOI:** 10.1371/journal.pone.0012786

**Published:** 2010-09-16

**Authors:** Brenda M. Murdoch, Michael L. Clawson, Samuel Yue, Urmila Basu, Stephanie McKay, Matthew Settles, Rossana Capoferri, William W. Laegreid, John L. Williams, Stephen S. Moore

**Affiliations:** 1 Department of Agricultural, Food and Nutritional Science, University of Alberta, Alberta, Edmonton, Canada; 2 Meat Animal Research Center, Agricultural Research Service (ARS), United States Department of Agriculture (USDA), Clay Center, Nebraska, United States of America; 3 Department of Veterinary Pathobiology, University of Illinois, Urbana, Illinois, United States of America; 4 Divisions of Animal Sciences, University of Missouri, Columbia, Missouri, United States of America; 5 Department of Biological Sciences, University of Idaho, Moscow, Idaho, United States of America; 6 IDRA Lab, Istituto Sperimentale Italiano, L. Spallanzani, Lodi, Italy; 7 Parco Tecnologico Padano, Polo Universitario, Lodi, Italy; Innsbruck Medical University, Austria

## Abstract

**Background:**

Classical bovine spongiform encephalopathy (BSE) is an acquired prion disease of cattle. The bovine prion gene (*PRNP*) contains regions of both high and low linkage disequilibrium (LD) that appear to be conserved across *Bos taurus* populations. The region of high LD, which spans the promoter and part of intron 2, contains polymorphic loci that have been associated with classical BSE status. However, the complex genetic architecture of *PRNP* has not been systematically tested for an association with classical BSE.

**Methodology/Principal Findings:**

In this study, haplotype tagging single nucleotide polymorphisms (htSNPs) within *PRNP* were used to test for association between *PRNP* haplotypes and BSE disease. A combination of Illumina goldengate assay, sequencing and PCR amplification was used to genotype 18 htSNPs and 2 indels in 95 BSE case and 134 control animals. A haplotype within the region of high LD was found to be associated with BSE unaffected animals (*p*-value = 0.000114).

**Conclusion/Significance:**

A *PRNP* haplotype association with classical BSE incidence has been identified. This result suggests that a genetic determinant in or near *PRNP* may influence classical BSE incidence in cattle.

## Introduction

Transmissible spongiform encephalopathies (TSEs), also known as prion diseases, are a group of mammalian neurodegenerative diseases that are invariably fatal and affect humans, ruminants, cats, and mink [1] (reviewed by [2]). TSEs are characterized by abnormal deposits of a protease-resistant isoform of the host genome encoded prion protein and are unique in that they can manifest through acquired, inherited, or sporadic origins [3]. At least three distinct bovine spongiform encephalopathies (BSEs) are known to afflict cattle [4]. The most common TSE of cattle is classical BSE with 190,542 cases reported in 26 countries (http://www.oie.int/eng/info/en_esbmonde.htm). Classical bovine spongiform encephalopathy is an acquired TSE that is most likely spread through the consumption of meat and bone meal contaminated with the infectious prion agent. Dietary exposure to products from BSE infected cattle is the suspected cause of the human TSE, variant Creutzfeldt-Jakob Disease (vCJD) [5]–[7]. Atypical BSE or BASE occurs in two forms, “H” and “L” [8], [9]. Approximately 51 atypical BSE cases have been reported worldwide (quote from Dr. Reg Butler, http://www.ibtimes.com/contents/20100318/cattle-disease-classical-bse-atypical-bse.htm).

The prion protein is essential for the development of TSE disease [10], and genetic variations in the prion gene (*PRNP*) have been associated with TSE susceptibility in humans [3], [11], sheep [12], [13], deer [14], [15], and cattle [16]–[19]. Two bovine *PRNP* alleles have been associated with susceptibility to classical BSE: a 23 base pair (bp) deletion within the promoter region and a 12 bp deletion within intron 1 [16]–[19]. However, the deletion alleles are not entirely independent of one another as there is high linkage disequilibrium (LD) between the two polymorphic sites in *Bos taurus* cattle populations [20]. This suggests that the possible effects of variations in the *PRNP* gene on incidence of classical BSE may be better understood if *PRNP* haplotypes were considered in testing for association with disease incidence. Moreover, *PRNP* haplotypes, containing one or both of the two insertion/deletion alleles, may have a stronger association with either susceptibility or resistance to classical BSE than if the indels are considering independently.

More than 390 polymorphisms have been described in a 25-kb region of chromosome 13 containing the *PRNP* gene [20], [21]. This chromosomal segment contains distinct regions of high and low LD that is conserved across many *Bos taurus* cattle populations [20]. The region of high LD includes the promoter region, exons 1 and 2, and part of intron 2 (6.7-kb) of the *PRNP* gene. Importantly, both the 23- and 12-bp indels that have been associated with classical BSE susceptibility are contained in this region of high LD. The remainder of *PRNP*, including the entire coding region has relatively low LD. To account for the genetic architecture of the *PRNP* gene, a set of haplotype tagging single nucleotide polymorphisms (htSNPs) has been described that efficiently define haplotypes within and across each of the LD regions. These htSNPs can be used to test for association between *PRNP* haplotypes and susceptibility to either classical or atypical BSE susceptibility [20], [22]. In this study, 18 htSNPs, including the 12-bp, and 23-bp indels were used to test *PRNP* haplotypes for an association with classical BSE in European Holstein cattle. Haplotypes associated with healthy control animals and classical BSE were identified.

## Results

The objective of this study was to test for association between *PRNP* haplotypes and BSE disease using a set of htSNPs that effectively tag haplotypes d within and across the *PRNP* locus. All htSNPs previously described by Clawson *et al.* [20] and the 23 bp and 12-bp indels described by Hill *et al.* [23] within the *PRNP* gene were genotyped in 330 European Holstein cows, of which 146 were BSE cases and 184 were controls. Single marker and haplotype analyses were performed using PLINK software [24]. Although many of these htSNPs had been previously genotyped [25] and tested for single marker associations with BSE disease status, the complete set of htSNPs and the 23 and 12-bp indels were not included in the earlier study, and therefore the haplotypes were not tested for association with BSE disease.

Initially 18 of the haplotype tagging SNPs, as well as the 23 and 12-bp indels, were tested independently, for an association with disease status. Three of the htSNPs were monomorphic in the sample set and therefore were not included in the haplotype analysis. A single SNP located at 13861 bp (based on NCBI accession number DQ457195) showed a significant association with disease incidence with a Bonferroni corrected *p* value of 0.027 and an odds ratio (OR) of 1.885 (Table 1). A significant association with htSNP 13861 and BSE incidence was also observed in an additional family based case control sample set described in Murdoch *et al.* [25] (Table S1). SNP 13861 which is location in intron 2 has not been identified as a regulatory region of *PRNP* [26]. Additionally, the T allele at the htSNP 4136 was over represented in control animals in the single marker association analysis (Table 1 and [25]), however, the association was not significant after correcting for the multiple testing. Further, in an additional family-based sample set described by Murdoch *et al.* [25], the frequency of htSNP 4136 was low (MAF<0.05) and was not found to have a significant association with disease status (Table S1).

**Table 1:** *PRNP* htSNPs and indel frequencies within BSE and case animals.

SNP ID	Allele 1	Frequency in BSE affected	Frequency in unaffected	Allele 2	*p-value* (uncorrected) for BSE association	*p-value* (corrected) for BSE association	Odds Ratio
*snp 248	C	0	0	T	NA	1	NA
*snp 449	G	0.290	0.327	T	0.393	1	0.838
*snp 1392	T	0.005	0.004	C	0.807	1	1.413
*snp 1567	T	0	0	C	NA	1	NA
*snp 1701	A	0.405	0.448	G	0.365	1	0.840
*snp 1783	A	0	0	G	Na	1	NA
*indel 23-bp*	I	0.290	0.361	D	0.110	1	0.722
*snp 3641	C	0.290	0.351	T	0.168	1	0.754
*snp 4136	T	0.047	0.106	C	0.024	0.48	0.419
*indel 1-2 bp*	I	0.342	0.417	D	0.104	1	0.726
*snp 4732*	A	0.310	0.250	G	0.182	1	1.350
*snp 4776	T	0.147	0.136	C	0.740	1	1.095
*snp 6811	T	0.021	0.011	A	0.397	1	1.900
*snp 8631	G	0.431	0.462	A	0.510	1	0.881
*snp 9162	C	0.006	0	T	0.233	1	NA
*snp 9786	C	0.447	0.485	T	0.426	1	0.859
*snp 13793*	G	0.483	0.402	A	0.097	1	1.387
*snp 13861*	C	0.574	0.417	G	0.001	0.027	1.885
*snp 13925*	G	0.067	0.062	C	0.820	1	1.096
*snp 17284*	A	0.111	0.061	G	0.130	1	1.920
*snp 20720	T	0.011	0.004	C	0.374	1	2.840
*snp 20957	T	0.058	0.063	C	0.807	1	0.907
*snp 21680	T	0.367	0.407	C	0.392	1	0.846

The SNP denoted by * was previously tested for an association with classical BSE and no significant associations were found (Murdoch *et al*. [25]). The presence of NA denotes that SNP was not analyzable due to the absence in the unaffected sample set.

**Figure 1:**
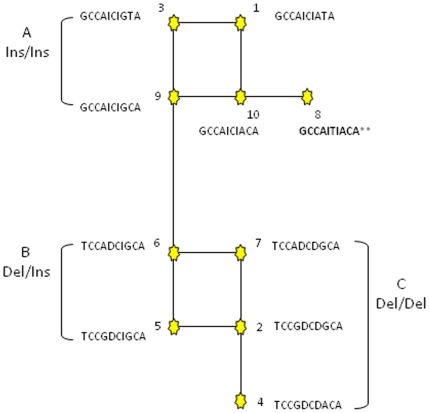
Median-joining network of haplotypes identified in the *PRNP* region of high LD. The median-joining network of Haplotypes SNP 449 | SNP 1392 | SNP 1576 | SNP 1701 | indel 23 | SNP 4136 | indel 12 | SNP 4732| SNP 4776| SNP 6811 A) Haplotypes with insertion alleles for both the 23 and 12-bp indels. ** denotes haplotype 8 within this haplotype block that is significantly association (*p* = 0.005) with unaffected BSE animals. B) Haplotypes with the deletion allele for the 23-bp indel and the insertion allele for the 12 bp-indel. C) Haplotypes with deletion alleles for both indels. Briefly, the haplotypes within the brackets only differ from the joining haplotype by one SNP. For example within bracket A haplotype 3 differs from haplotype 9 at SNP 4776, where haplotype 3 is a T and 9 is a C.

The genetic architecture of *PRNP* that has regions of high and low LD, and is conserved across many *Bos taurus* populations, was taken into consideration in the haplotype-case association analysis. HtSNPs previously described by Clawson *et al.* [20] were used to define the haplotypes present: nine htSNPs are in the low LD region, and ten htSNPs including both the 23 and 12-bp indels are in the high LD region (Figure 1). A segment of *PRNP* within low LD region, containing htSNPs 13793, 13861 and 13925, is rich in GC content and contains a multi- G indel. Although 330 samples were sequenced, 101 of the samples did not yield reliable sequence quality and thus genotypes for this region; htSNPs 13793, 13861 and 13925. These 101 samples were not analyzed further and a reduced sample set of 95 cases and 134 controls was used for testing the association between haplotype and BSE status. No haplotype associations with BSE disease status were found within the region of low LD. However, within the region of high LD, one haplotype (Table 2, haplotype 8) was significantly over represented in unaffected animals (*p* = 0.005). This haplotype has been previously identified [20] and contains insertion alleles for both indel 23 and 12, and is tagged by the 4136 htSNP (Figure 1, Table 2).

**Table 2:** Analysis of network one haplotype block consisting of 8 htSNPs and 2 indels.

Haplotype											Frequency of BSE affected	Frequency of unaffected	*p* value
1	G	C	C	A	I	C	I	A	T	A	0.132	0.130	0.968
2	T	C	C	G	D	C	D	G	C	A	0.542	0.481	0.359
3	G	C	C	A	I	C	I	G	T	A	0.0185	0.0156	0.871
4	T	C	C	G	D	C	D	A	C	A	0.0629	0.0167	0.106
5	T	C	C	G	D	C	I	G	C	A	0.0135	0.0223	0.603
6	T	C	C	A	D	C	I	G	C	A	0.0355	0.0472	0.652
7	T	C	C	A	D	C	D	G	C	A	0.0561	0.0491	0.817
8	G	C	C	A	I	T	I	A	C	A	0.0332	0.123	0.00504**
9	G	C	C	A	I	C	I	G	C	A	0.0554	0.0934	0.256
10	G	C	C	A	I	C	I	A	C	A	0.0506	0.0214	0.272

Haplotype block for SNP 449 | SNP 1392 | SNP 1576 | SNP 1701 | indel 23 | SNP 4136 | indel 12 | SNP 4732| SNP 4776| SNP 6811 ** denote significance of *p*<0.01.

The association between haplotype 8 and BSE (shown in Figure 1) was tested in comparison with haplotypes formed from the 23- and 12-bp indels alone. The alleles at these two indels were tightly linked and the haplotype defined by the insertion allele of the 23-bp indel, and the deletion allele of 12-bp indel (ID) was not observed, while the deletion/insertion (DI) haplotype was at low frequency in both the cases and controls. The deletion/deletion (DD) haplotype was the most frequent haplotype in both case and control populations, with a significantly higher frequency in the cases than controls (*p* = 0.032). Conversely, the insertion/insertion (II) haplotype was at significantly higher frequency in the controls than in the cases (*p* = 0.038) (see Table 3).

**Table 3:** Frequency of 23-bp and 12-bp indel haplotype block.

Haplotype	Frequency in BSE affected	Frequency in unaffected	*p* value
II	0.283	0.409	0.038*
DI	0.0588	0.0682	0.764
DD	0.658	0.523	0.032*

*Denotes significance of *p*<0.05.

Although, the 23 and 12-bp indel haplotype was shown to be associated with BSE disease the level of significance was lower than for haplotype 8, which also contained htSNP 4136, suggesting an effect of the haplotype and that the indels alone are not responsible for the association observed (see Figure 1). The haplotype which was significantly associated with the absence of disease contained the insertion alleles of the 23 and 12-bp indels in addition to the T allele of htSNP 4136. The T allele further separates the insertion/insertion haplotype into two haplotypes (Table 4), one of which is more tightly associated with classical BSE resistance than the haplotype defined by the insertion/insertion alleles alone (Tables 2, 3, and 4).

**Table 4:** Haplotype analysis results with reduced htSNP set.

Locus		Haplotype			Frequency of BSE affected	Frequency of unaffected	p value
1	A	I	T	I	0.0373	0.173	0.000114***
2	A	I	C	I	0.246	0.236	0.854
3	A	D	C	I	0.0417	0.0448	0.905
4	G	D	C	I	0.0173	0.0234	0.731
5	A	D	C	D	0.076	0.0688	0.833
6	G	D	C	D	0.582	0.454	0.0472*

Haplotype defined by the alleles of SNP 1701 | indel 23 |SNP 4136 | indel 12 *** denotes significance of *p*<0.001.

The alleles of htSNP 449 and those of the 23-bp indel were found to be tightly linked in this study (Tables 1 and 3), whereas the three htSNPs (1392, 1567, and 6811) had very low minor allele frequencies in both BSE cases and controls (MAF<0.05) making the power of testing the association of these loci with disease status low. Therefore, the htSNPs with a low minor allele frequency were excluded from further haplotype analysis. Haplotypes with and without the alleles of htSNP 449 and the 23-bp indel were independently tested for association with BSE (Table S2). A haplotype defined by a minimal set of four SNP alleles (1701/23-bp indel/4136/12-bp indel) was found to be significantly associated with BSE status (Table S2 and Table 4). The haplotype characterized by allele A at SNP 1701, insertion allele at the 23-bp indel, T at SNP 4136 and insertion in the 12-bp indel was significantly over represented in unaffected animals (*p* = 0.000114, see Table 4). Whereas the haplotype characterized by the G allele at SNP 1701, deletion at the 23-bp indel, C allele at SNP 4136 and deletion in the 12-bp indel was over represented in BSE affected animals although the level of significance was lower (*p* = 0.0472).

## Discussion

A haplotype defined by the alleles of four polymorphisms in the region of high LD within the *PRNP* gene was found to be associated with reduced incidence classical BSE. The haplotype includes insertion alleles at the 23 and 12-bp indels which have previously been shown to be associated with classical BSE [14]–[17]. The haplotypes also contains htSNP 1701 and 4136, the latter showing the strongest individual association with BSE, with the “T” allele associated with control in this study. The haplotype is in a region of high LD and the extent of this haplotype is unknown. Nonetheless, this finding indicates that a genetic determinant in or near the region of high LD in the *PRNP* has an effect on resistance or susceptibility to classical BSE.

The 23 and 12-bp indel alleles of *PRNP* were tested independently of the htSNPs for an association with classical BSE, as these loci have been associated with BSE status in previous studies [16]–[18]. In the present study, a haplotype defined by the insertion alleles of both 23- and 12-bp indels (the insertion/insertion haplotype) was significantly associated with unaffected animals (*p* = 0.038), and the deletion/deletion haplotype was associated with BSE affected animals (*p* = 0.032), which is in agreement with the previous reports. However this association had a lower level of significance than haplotype 8 which included the 4136 htSNP. Both the indels contain transcription factor binding sites: the 23-bp indel insertion allele contains a repression factor RP-58 binding site and the insertion allele of the 12-bp indel contains a SP-1 binding site [26]. The presence of the 12-bp insertion allele may disrupt a coordinated regulation of the prion gene [27]. In addition, different indel haplotypes within the *PRNP* gene have been shown to have different levels of expression in cell culture, with higher expression associated with the deletion/deletion allele compared to the insertion/insertion allele [17]. However, it is not known if the level of expression *per se* has an effect on BSE susceptibility.

**Figure 2:**
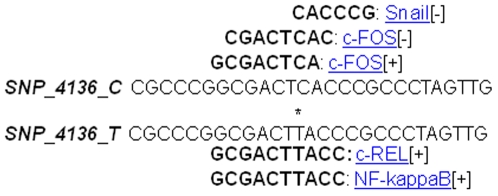
Sequence for haplotype tagged SNP 4136 [C/T] binding sites. The * denotes the position of htSNP 4136 and where the alleles differ.

*PRNP* htSNP 4136 is located in the promoter region exactly 14 bases upstream from exon 1 (GenBank file DQ457195). It has been shown that expression of the bovine prion protein gene requires interaction between the promoter and the first intron [25]. It was therefore possible that this htSNP had an effect on promoter activity. Thus bioinformatic analysis of sequence containing the htSNP 4136 [C/T] was performed using ConSite (http://asp.ii.uib.no:8090/cgi-bin/CONSITE/consite/). This analysis identified potential changes in transcription factor binding sites: the sequence with the more common C allele, which is associated with disease, has a putative c-Fos binding site, whereas, the T allele has a putative NF-kappaB binding site (see Figure 2 and Table 5). c-Fos can suppress the expression of c-Jun/ATF2 which promotes neuronal apoptosis [28], while NF-KappaB may have a role in prion diseases through the inflammatory response [29]–[32]. It is not know if these variations are biologically relevant or have an effect on the course of BSE infection. Investigation of the effect of transcription factor binding activities in the promoter region of the bovine *PRNP* from a variety of different haplotype sequences found in the *Bos taurus* population would be interesting.

**Table 5:** Putative transcription binding sites for htSNP 4136 C and T allele.

SNP	Transcription factor	Sequence	Score	Strand
	c-FOS	GCGACTCA	7.051	+
4136_C	c-FOS	CGACTCAC	7.633	−
	Snail	CACCCG	6.063	−
4136_T	NF-kappaB	GCGACTTACC	7.333	+
	c-Rel	GCGACTTACC	6.623	−

The htSNP 13861 was significantly associated with BSE disease incidence (Bonferroni corrected *p* value 0.027). A significant association (Bonferroni corrected *p* value 0.016) with BSE status was also found in an additional samples set of related BSE cases and controls (Table S1). The htSNP 13861 is within the second intron and immediately 5′ of a indel that consists of alleles with multiple G's and although that was difficult to resolve by sequencing may serve as a marker for the multi-G indel. Although this region of the *Bos taurus PRNP* gene has not been previously identified as containing regulatory elements [25] many purine-rich regions have been shown to have regulatory function. Specifically the GGG triplet is a common splicing control element (review by [33]). Further, heterogeneous nuclear ribonucleoprotein (hnRNP) A2/B1, a gene that is associated with pre-mRNA processing, has been previously identified as an interactive partner of the prion protein [34]. It is not known if the [C/G] htSNP 13861 which is located proximally to a multiple G indel is associated with *PRNP* splicing control or if there may be a relationship that with BSE disease.

This study further defines the association of the 23 and 12-bp indel with classical BSE resistance previously described [17]–[19]. Specifically when htSNPs 1701 and 4136 are included in the haplotype the significance of the association increases from *p* = 0.038 to *p* = 0.00011. The association of this haplotype with classical BSE has not been previously reported. However, in a previous study [22], a haplotype in the low LD region of *PRNP* was associated with atypical BSE (BASE). The haplotype associated with atypical BSE was not found to be significantly associated with classical BSE in this study. This is consistent with a previous report [35] which suggested that variation in the *PRNP* gene that may influence classical BSE susceptibility are not associated with other TSE in cattle.

While the origins of classical and atypical BSEs are not known they, in common with all TSEs, require the prion protein for the manifestation of disease. The present study and others have shown that variations in *PRNP* are associated with either incidence of classical or atypical BSE [16]–[19], [5], [22], although the extensive LD within some regions of bovine *PRNP* make it difficult to determine which, if any of the variations have a functional role. Variations in PRNP are important factors for understanding susceptibility of BSE and potentially managing bovine TSE in cattle. The results reported here better define the variations within the bovine *PRNP* gene that have an effect on susceptibility or resistance to BSE and contribute to an improved definition of the genetic factors involved in the disease.

In conclusion a *PRNP* haplotype was identified that associated with classical BSE incidence. The haplotype is contained within known region of high linkage disequilibrium in *Bos taurus* cattle. The haplotype may be linked with a genetic determinant in or near *PRNP* that influences classical BSE susceptibility.

## Materials and Methods

This study used DNA, extracted from blood samples of 333 Holstein cows from the UK, of which 149 were BSE cases and 184 were unaffected controls. BSE positive cattle were examined by qualified veterinarians and their BSE status was subsequently confirmed post-mortem by histology (by the Veterinary Laboratories Agency, New Haw, Surrey, UK). Control animals did not exhibit any BSE symptoms at the time of collection and were age matched with BSE cases from the same farm. Included in the control group were 15 BSE negative animals, confirmed by post-mortem histology. The case and control blood samples were all collected by the UK Veterinary Investigation Service and local veterinary practitioners in the mid 1990s in Southern England, at the peak of the BSE epidemic. The family-based samples set consisted of 302 BSE affected and 179 unaffected half-sib Holsteins from six sire families. All the BSE affected and unaffected cattle within one family were paternal half sibs from the designated sire but with different dams. DNA samples were not available from any of the sires or dams.

Genomic DNA was isolated from blood using a high salt phenol/chloroform extraction method as described by Sherman *et al.* [36]. Genotyping was performed using an oligonucleotide pool assay (OPA) as described by McKay and others [37]. This OPA comprises SNPs covering all chromosomes and included 15 of the 19 *PRNP* htSNPs reported by Clawson *et al.* [20]. An Illumina GoldenGate assay [38] was performed using the OPA and genotypes determined using an Illumina BeadScan (Illumina Inc., San Diego, CA) and the Illumina BeadStudio software. The four *PRNP* htSNPs, in the *PRNP* promoter region that were not present in the OPA were genotyped by PCR amplification of the region and Sanger sequencing as described by Clawson *et al.* [20], [39]. Genotypes for the 12 and 23-bp indel were identified by PCR and the alleles were resolved on a 3% agarose gel stained with ethidium bromide. The multiplex PCR reaction which included 10 µl of genomic DNA, forward primer CTCGGTTTTACCCTCCTGG and the reverse primer GGCTAGATTCCTACACACCACC for the 12-bp indel, the forward primer CCTGATTTTCAAGTCCTCCCAG and the reverse primer TTATGCCCATGAATTGTGTAGGC for the 23-bp indel was carried out following the manufacturer's protocols (Accuprime *Taq* DNA polymerase system, Invitrogen). The thermocycling conditions were: an initial denaturation of 2 minute at 94°C, followed by thirty cycles at 94°C for 2 min, 54°C for 30 sec. and 68°C for 1 min.

PLINK v1.04 [23] was used to phase haplotypes and perform all of the statistical analysis. The data from the case-control sample set were analyzed using the basic case-control association (χ^2^) test and the haplotype-based association test was used for all of the haplotype analysis.

## Supporting Information

Table S1 PRNP htSNPs and indel frequencies within family BSE and case animals.
(0.16 MB RTF)

Table S2 Results of comparative haplotype analysis with different combinations. The first table includes both htSNP 449 and indel 23; whereas the following tables include either htSNP 449 or indel 23.
(0.16 MB RTF)
