# Supplementary material for: PRNP Haplotype Associated with Classical BSE Incidence in European Holstein Cattle
Source: PLoS One. 2010 Sep 16;5(9):e12786. doi: 10.1371/journal.pone.0012786 (PMC2940907; doi:10.1371/journal.pone.0012786)
Supplement: Table S1 — PRNP htSNPs and indel frequencies within family BSE and case animals. (0.16 MB RTF) [file pone.0012786.s001.rtf]

Table S1. PRNP htSNPs and indel frequencies within family BSE and case animals 
SNP ID	Allele 1	Frequency in BSE affected	Frequency in unaffected	Allele 2	p-value (uncorrected) for BSE association	p-value (corrected) for BSE association	Odds Ratio	
*snp 248	C	0	0	T	0.067	1	NA	
*snp 449	G	0.193	0.266	T	0.010	1	0.660	
*snp 1392	T	0	0	C	0.844	1	NA	
*snp 1567	T	0	0	C	0.699	1	NA	
*snp 1701	A	0.289	0.310	G	0.525	1	0.907	
*snp 1783	A	0	0	G	0.065	1	NA	
indel 23-bp 	I	0.210	0.279	D	0.016	1	0.687	
*snp 3641	C	0.219	0.223	T	0.891	1	0.978	
*snp 4136	T	0.045	0.054	C	0.600	1	0.838	
indel 12-bp 	I	0.281	0.335	D	0.080	1	0.774	
snp 4732	A	0.207	0.265	G	0.055	1	0.722	
*snp 4776	T	0.117	0.197	C	0.002	0.039	0.543	
*snp 6811	T	0	0	A	0.608	1	NA	
*snp 8631	G	0.380	0.418	A	0.253	1	0.852	
*snp 9162	C	0.	0	T	0.287	1	NA	
*snp 9786	C	0.739	0.715	T	0.437	1	1.128	
snp 13793	G	0.503	0.381	A	0.014	1	1.644	
snp 13861	G	0.460	0.622	C	0.001	0.016	0.518	
snp 13925	G	0.179	0.117	C	0.055	1	1.656	
snp 17284	A	0.051	0.081	G	0.178	1	0.613	
*snp 20720	T	0	0	C	0.065	1	NA	
*snp 20957	T	0.188	0.171	C	0.521	1	1.120	
*snp 21680	C	0.380	0.395	T	0.663	1	0.939	
The SNP denoted by * was previously tested for an association with classical BSE and no significant associations were found (Murdoch et al. [25]).  The presence of NA denotes that SNP was not analyzable due to the absence in the unaffected sample set.
